# Transcriptome Landscapes of Salt-Susceptible Rice Cultivar IR29 Associated with a Plant Growth Promoting Endophytic *Streptomyces*

**DOI:** 10.1186/s12284-023-00622-7

**Published:** 2023-02-04

**Authors:** Worarat Kruasuwan, Karan Lohmaneeratana, John T. Munnoch, Wanwipa Vongsangnak, Chatchawan Jantrasuriyarat, Paul A. Hoskisson, Arinthip Thamchaipenet

**Affiliations:** 1 Department of Genetics, Faculty of Sciences, Kasetsart University, Bangkok, Thailand; 2 Strathclyde Institute of Pharmacy and Biomedical Sciences, University of Strathclyde, Glasgow, UK; 3 Department of Zoology, Faculty of Sciences, Kasetsart University, Bangkok, Thailand; 4 Omics Center for Agriculture, Bioresources, Food and Health, Kasetsart University (OmiKU), Bangkok, Thailand; 5 Present Address: Siriraj Long-Read Lab (Si-LoL), Division of Medical Bioinformatics, Research Department, Faculty of Medicine Siriraj Hospital, Mahidol University, Bangkok, Thailand

**Keywords:** *Streptomyces*, *Oryza sativa* L. cv. IR29, Plant–microbe interaction, ACC deaminase, Salt tolerance

## Abstract

Plant growth-promoting endophytic (PGPE) actinomycetes have been known to enhance plant growth and mitigate plant from abiotic stresses via their PGP-traits. In this study, PGPE *Streptomyces* sp. GKU 895 promoted growth and alleviated salt tolerance of salt-susceptible rice cultivar IR29 by augmentation of plant weight and declined ROS after irrigation with 150 mM NaCl in a pot experiment. Transcriptome analysis of IR29 exposed to the combination of strain GKU 895 and salinity demonstrated up and downregulated differentially expressed genes (DEGs) classified by gene ontology and plant reactome. *Streptomyces* sp. GKU 895 induced changes in expression of rice genes including transcription factors under salt treatment which involved in growth and development, photosynthesis, plant hormones, ROS scavenging, ion transport and homeostasis, and plant–microbe interactions regarding pathogenesis- and symbiosis-related proteins. Taken together, these data demonstrate that PGPE *Streptomyces* sp. GKU 895 colonized and enhanced growth of rice IR29 and triggered salt tolerance phenotype. Our findings suggest that utilisation of beneficial endophytes in the saline fields could allow for the use of such marginal soils for growing rice and possibly other crops.

**Supplementary Information:**

The online version contains supplementary material available at 10.1186/s12284-023-00622-7.

## Background

Salinity is a harsh environmental factor limiting the growth and productivity of several crop plants. Over 20% of total irrigated land has been damaged by salinity and nearly 800 million hectares (more than 6%) of the world’s total land is saline soil (Pitman and Läuchli 2002). Considering a very small number of halophyte species, crops are mostly salt-sensitive. In Thailand, there are 2.3 million hectares, mostly in the northeast part of Thailand where saline soil influences the low yield of commercial crop plantations (Arunin and Pongwichian 2015). Rice (*Oryza sativa* L.) is one of the most important food crops in the world, however, its growth, development and productivity are affected by adverse conditions including saline soil.

Salinity adversely affects plant growth by causing high internal Na^+^ accumulation and disturbing the expression of several genes involved in growth and development. Plants cope with salinity by triggering signal cascades which result in the activation of transcription factors (TFs), eventually leading to regulation of salt tolerance gene expression (Gupta and Huang 2014). For instance, overexpression of abscisic acid (ABA)-responsive transcription factor (*OsNAC45*) improved salt tolerance and may regulate the expression of two specific genes, plasma membrane protein 1 (*OsPM1*) and late embryogenesis abundant (*OsLEA3-1*) in rice (Zhang et al. 2020). One option to overcome the severe effect of salinity on plant growth is to genetically modify plants, however, whilst targeting particular phenotypes through genome editing and multi-omics approaches have been successful, they are rarely used in the field due to environmental concerns and biosafety regulation (Linh et al. 2012; Raza et al. 2022). Alternatively, beneficial microbes associated with plants such as endophytes have been reported to enhance growth and alleviate stress tolerance of plants under severe conditions (Fan et al. 2020; Ganie et al. 2022). Plant growth-promoting endophytes (PGPE) influence plant growth and environmental stress tolerance via direct and indirect mechanisms including production of plant hormones, antioxidants, compatible solutes, phosphate solubilization, nitrogen fixation, and secretion of 1-aminocyclopropane-1-carboxylate (ACC) deaminase to reduce ethylene levels in plants (Etesami and Beattie 2018; Narsing Rao et al. 2022).

PGPEs such as *Brachybacterium*, *Enterobacter*, *Paenibacillus*, *Pseudomonas,* and *Streptomyces* have been shown to alleviate date palm, musli, sugarcane, and rice from salt stress by decreasing the accumulation of sodium ions and lowering oxidative stress as well as ethylene emission (Yaish et al. 2015; Barnawal et al. 2016; Kruasuwan and Thamchaipenet 2018; Jaemsaeng et al. 2018; Yoolong et al. 2019). Generally, salinity induced high activities of ACC synthase (ACS) and ACC oxidase (ACO) in plants and subsequently produced high levels of ethylene (Nascimento et al. 2018). However, plant ethylene can be reduced by certain plant-associated bacteria that possess an enzyme ACC deaminase by converting ACC to ammonia and α-ketobutyrate and subsequently reduced level of stress ethylene (Glick 2015). Genes expression profiles during beneficial bacteria and plant interactions under both normal and adverse conditions have been characterized; for example, rhizospheric *Arthrobacter nitroguajacolicus* enhanced salt tolerance of wheat plantlets by upregulation the expression of phenylpropanoid genes which related to lignin biosynthesis, antioxidant activity, and response to various types of plant pathogens and abiotic stresses (Safdarian et al. 2019). *Azospirillum brasilense* induced the expression of nutrient acquisition and cell cycle associated genes in wheat (Camilios-Neto et al. 2014), rhizospheric *Bacillus amyloliquefaciens* triggered systemic salt tolerance in Arabidopsis and rice by increasing plant biomass and stimulating genes involved in photosynthesis, auxin, SOS scavenging, Na^+^ translocation, and osmoprotectant (trehalose and proline) synthesis (Liu et al. 2017; Chauhan et al. 2019). Therefore, a thorough study of plant–microbe interactions would allow to elucidate the mechanisms induced by PGPEs, which enable plant salt-stress alleviation and consequently, growth promotion.

Indica rice cultivar IR29, a salt-susceptible high-yielding cultivar, was mostly used as rice model for several aspects regarding salinity. Previously, transcriptome, translatome, and DNA methylation in response to salt stress focusing mainly on the physiological aspects were performed in IR29 comparing to salt-tolerant rice cultivars (Razzaque et al. 2017; Ferreira et al. 2015; Li et al. 2018). However, there are less reports on transcriptome profiling of genes involved in salt tolerance during PGPE-IR29 interactions. In this work, endophytic *Streptomyces* sp. GKU 895 isolated from sugarcane revealed PGP-traits and enhanced growth of sugarcane (Kruasuwan and Thamchaipenet 2016) was evaluated its effect on the growth of rice cultivar IR29 under salinity (150 mM NaCl) condition. Moreover, transcriptomic profiling of rice genes responding to salinity stress during the interaction with *Streptomyces* sp. GKU 895 was characterized and supported the potential for beneficial effects of endophytes to enhance salt tolerance in rice.

## Results

### Endophytic *Streptomyces* Promoted Growth and Alleviated Salt Stress in Rice

**Fig. 1:**
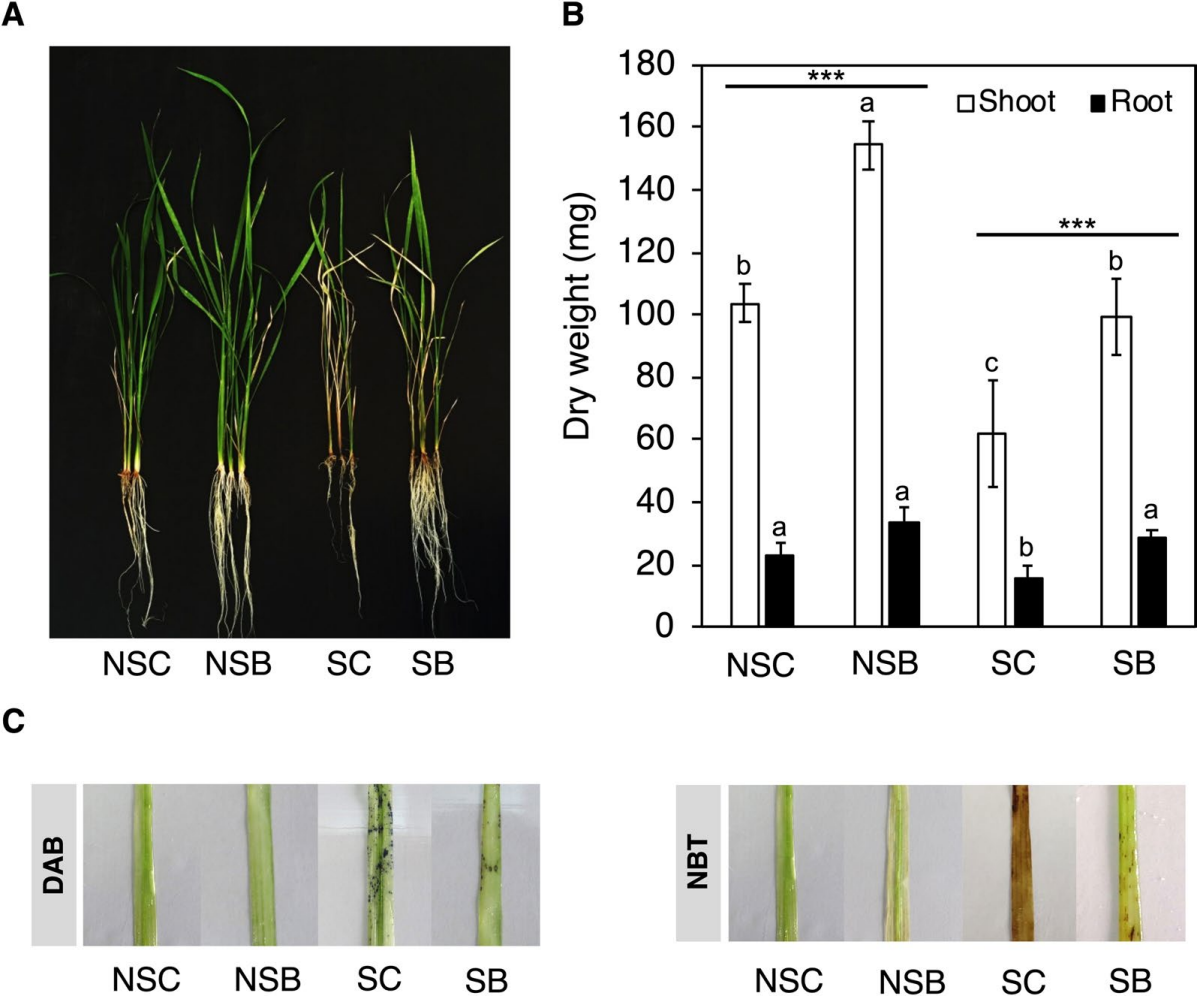
Effect of salinity and PGPE *Streptomyces* sp. GKU 895 inoculation on plant growth (**A**), biomass (**B**), and DAB and NBT straining of leaves (**C**) of salt-susceptible rice cultivar IR29 (*Oryza sativa* L. cv. IR29). NSC, non-salt control (0 mM NaCl); NSB, non-salt GKU 895 inoculation; SC, salt-stress control (150 mM NaCl); SB, salt-stress GKU 895 inoculation

Without salinity, inoculation of *Streptomyces* sp. GKU 895 (NSB) promoted the growth of rice cultivar IR29 by increment of root and shoot biomass at 50% and 49%, respectively when compared to the uninoculated plants (NSC); whereas with salinity, root and shoot biomass of GKU 895-inoculated rice (SB) increased by 82% and 61%, respectively when compared to the uninoculated plants (SC) (Fig. 1a, b, Additional file 1: Table S1). Superoxide and hydrogen peroxide (i.e., reactive oxygen species, ROS) were abundantly detected in SC leaves, while SB leaves was lower (Fig. 1c). Data show that the antioxidant produced by *Streptomyces* sp. GKU 895 is involved in its growth and salt tolerance promotion in rice IR29.

### Analysis of Differentially Expressed Genes (DEGs) of Rice Treatments

**Fig. 2:**
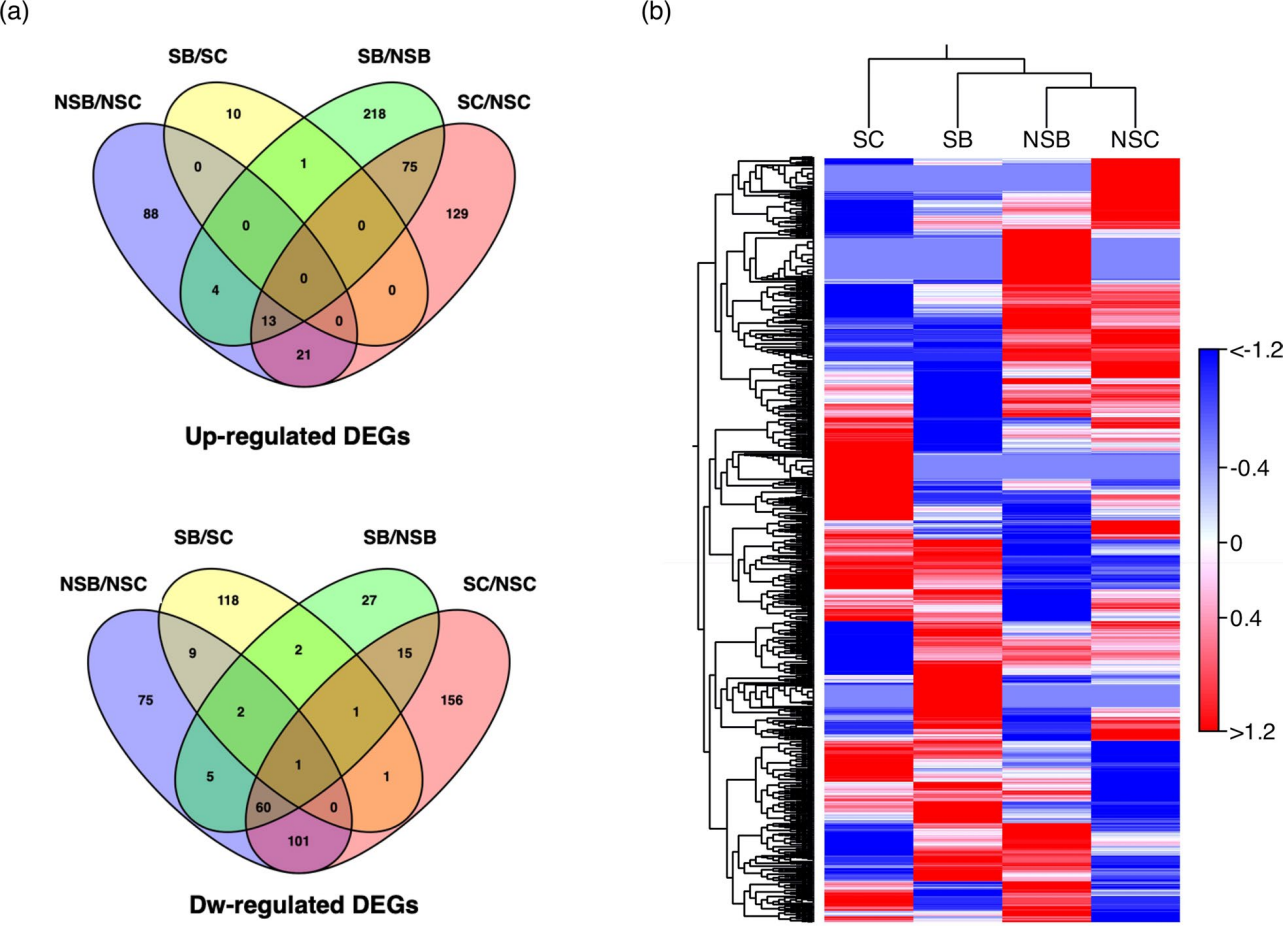
Up and downregulated differentially expressed genes (DEGs) of salt-susceptible rice cultivar IR29 (*Oryza sativa* L. cv. IR29) in response to *Streptomyces* sp. GKU 895 with and without salinity; Venn diagrams analysis (**a**) and Hierarchical cluster analysis (**b**). NSC, non-salt control (0 mM NaCl); NSB, non-salt GKU 895 inoculation; SC, salt-stress control (150 mM NaCl); SB, salt-stress GKU 895 inoculation

Ion Torrent generated approximately 1.9 Mbp of rice transcriptome data with 96-bp average read lengths of sequenced data from 4 treatments namely, non-salt control (NSC), non-salt GKU 895 inoculation (NSB), salt-stress control (SC), and salt-stress GKU 895 inoculation (SB). Approximately 78–85% of reads were mapped to the rice genome (*Oryza sativa* IRGSP-1.0; BioProject number PRJDB1747) (Additional file 1: Table S2). DEGs were then compared in pairs of NSB/NSC, SB/SC, NSC/SC, and NSB/SB (FDR < 0.05, FC ≥ 1.5 and ≤ 1.5 for up and downregulation, respectively). The mapped reads between NSB/NSC, SB/SC, SC/NSC, and SB/NSB were assembled to result in significant DEGs of 381, 147, 574, and 424, respectively. Amongst them, 126/253, 11/134, 238/335, and 311/113 DEGs were up/downregulated expression in NSB/NSC, SB/SC, SC/NSC, and SB/NSB conditions, respectively (Additional file 2: Table S3). The exclusive or common overlapping transcripts between up and downregulated DEGs of all treatments were plotted (Fig. 2a). The results showed no upregulated DEGs are shared in both NSB/NSC and SB/SC conditions, whereas 75 DEGs were found to overlap in SB/NSB and SC/NSC conditions suggesting that a common regulatory pathway of rice IR29 under salt treatment was activated by *Streptomyces* sp. GKU 895. Downregulated DEGs revealed that 9 DEGs were shared in non-salt (NSB/NSC) and salt-stress (SB/SC) treatments; and 15 DEGs were commonly found in salt-stress conditions with (SB/NSB) or without GKU 895 (SC/NSC) (Fig. 2a). Corresponding with transcriptional profile analysis by hierarchical clustering, the non-salt treatments, NSC and NSB, were distributed in the same clade, while the salt-stress treatments, SB and SC, were separated into distinct clades (Fig. 2b).

### Functional Categorization of DEGs

Based on gene ontology (GO) analysis of the common 75 upregulated DEGs between SB/NSB and SC/NSC, only one GO category was altered which was involved in cellular nitrogen compound metabolic process (GO: 0034641). But there are no enriched GO terms of biological processes in common downregulated DEGs (hypergeometric test *p* < 0.05, FDR < 0.05) (Additional file 3: Table S4). Moreover, an enrichment analysis of DEGs in each treatment identified 3 (NSB/NSC), 2 (SB/SC), 20 (SC/NSC), and 37 (SB/NSB) GO terms of biological processes (Additional file 4: Table S5). These data indicated that a greater number of biological processes GO terms were affected by *Streptomyces* sp. GKU 895 under salt-stress condition (SB/NSB). GO term associated cellular component biogenesis (GO: 004485) was increased in inoculated rice compared to uninoculated control (NSB/NSC). Conversely, salinity enhanced some genes belonging to GO categories related to translation (GO: 0006412) and cellular biosynthetic process (GO: 0044249) in downregulated DEGs of SC/NSC conditions. However, GO terms relating to aromatic amino acid associated metabolic processes (GO: 0009072) and ion transport (GO: 000681) were enriched in upregulated DEGs in GKU 895 inoculated rice IR29 under salt stress (SB/NSB) (Additional file 4: Table S5).

Metabolic pathways regarding photorespiration and calvin cycle were downregulated in salt-stressed rice (SB/NSB) inoculated rice (NSB/NSC), as well as in salt-stress control (SC/NSC) conditions; but these pathways were upregulated in the presence of salt-stressed inoculated rice (SB/SC). The SNAC1 transcription network involved in drought and salinity tolerance was found to be upregulated in salt-stressed rice (SC/NSC) and rice inoculated with GKU 895 (NSB/NSC), while genes involved in phenylpropanoid and suberin biosynthesis were only induced in salt-stressed rice inoculated with *Strepromyces* sp. GKU 895 (SB/NSB) (Additional file 5: Table S6).

### DEGs Annotated as Transcription Factors (TFs)

**Fig. 3:**
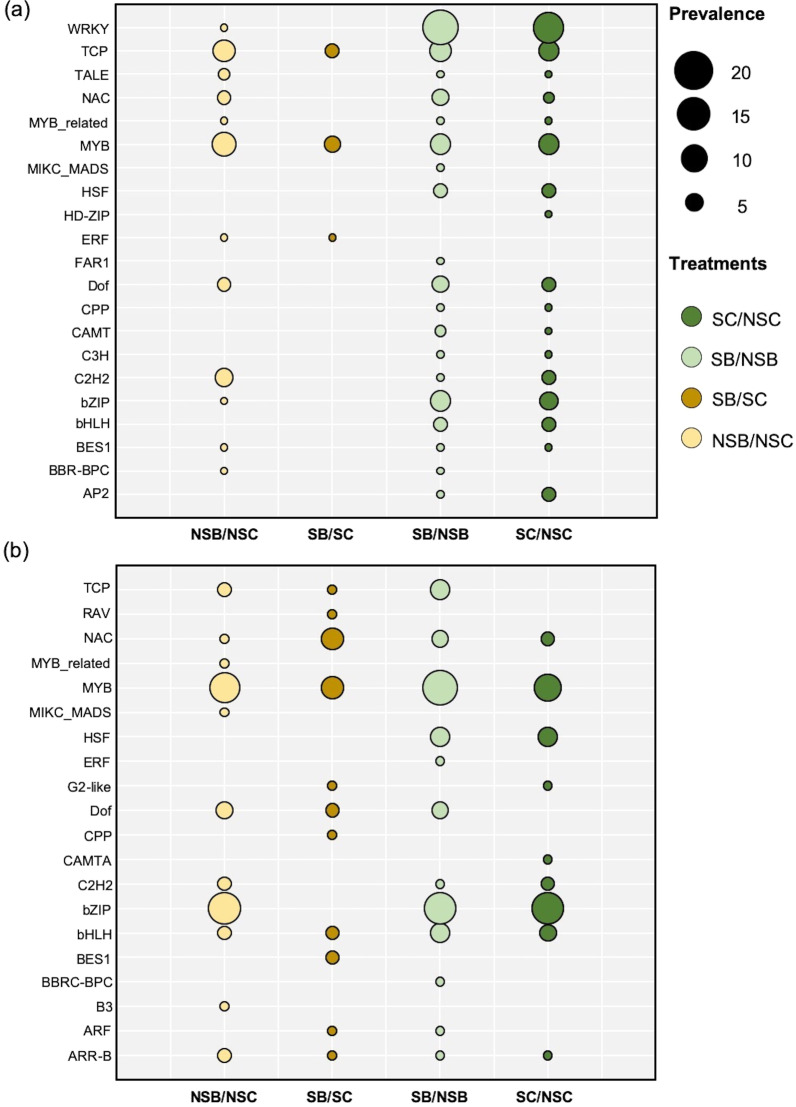
Family of transcription factors displayed up (**a**) and downregulated (**b**) differentially expressed genes (DEGs) of salt-susceptible rice cultivar IR29 (*Oryza sativa* L. cv. IR29) in responded to *Streptomyces* sp. GKU 895 with and without salinity. NSC, non-salt control (0 mM NaCl); NSB, non-salt GKU 895 inoculation; SC, salt-stress control (150 mM NaCl); SB, salt-stress GKU 895 inoculation

A total of 159 and 132 of differentially expressed TFs distributing into 21 and 20 families were respectively predicted in up and downregulated DEGs (Fig. 3, Additional file 6: Table S7). Of these, ARR-B, bHLH, MYB, NAC, and TCP TF families were commonly expressed across up and downregulated DEGs. Of upregulated DEGs, WRKY TFs responding to salt stress (*OsWRKY24* and *OsWRKY57*) were predominantly represented in salt-stressed rice with (SB/NSB) or without (SC/NSC) *Streptomyces* sp. GKU 895 inoculations, whereas the ethylene-responsive TF ERF (*OsERF12*) was only enriched in salt-stressed inoculated rice (SB/SC). In downregulated DEGs, bZIP and MYB were notably annotated in all treatments except SB/SC for bZIP. CAMTA (LOC_Os03g09100) responding to abiotic stress was only found in salt-stressed rice (SC/NSC), while salt-stress inoculated plants (SB/SC and SB/NSB) regulated expression of a key regulator of cell proliferation ARF (LOC_Os01g70270) (Fig. 3).

### Endophytic *Streptomyces* Modulate Expression of Salt-Stress Responsive Genes

**Fig. 4:**
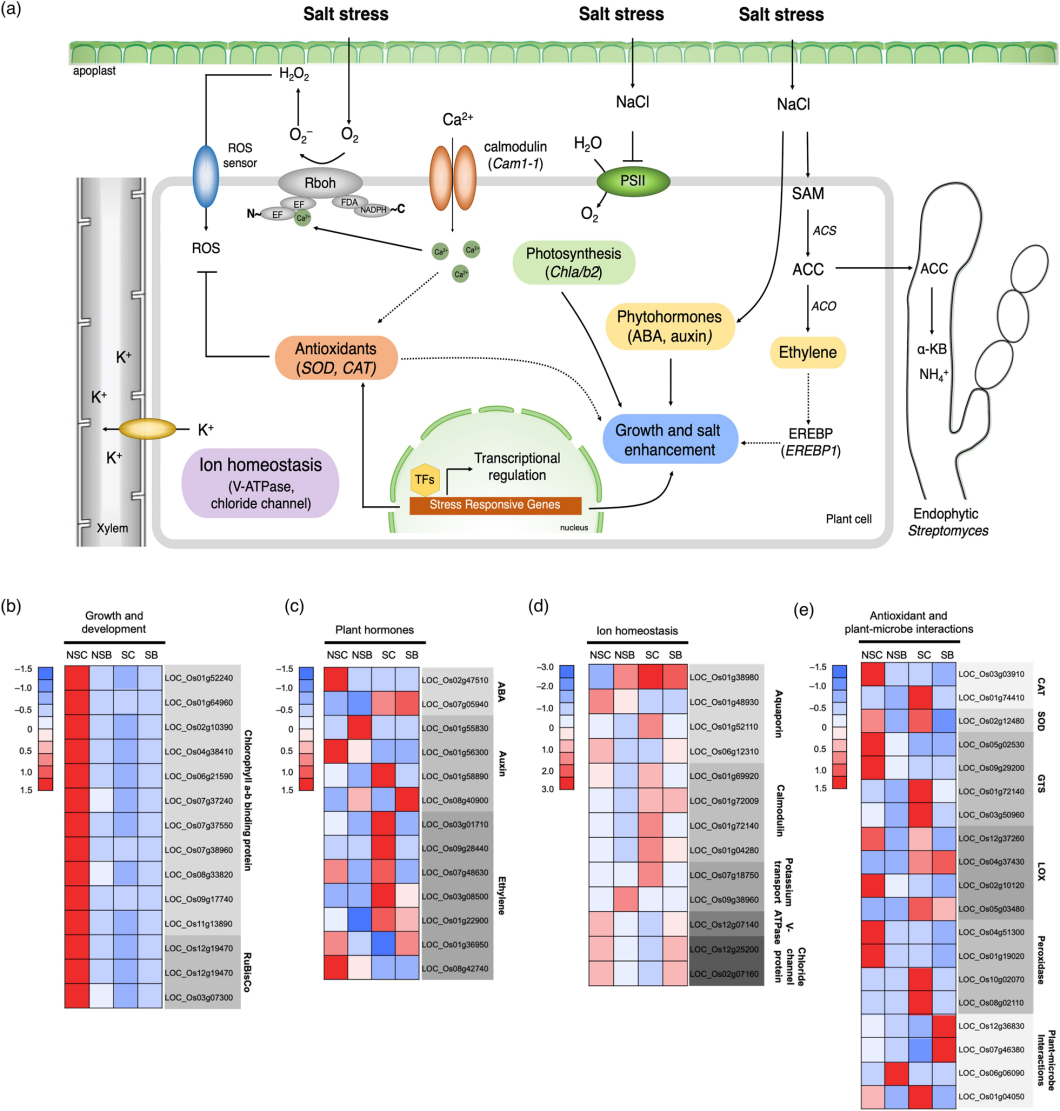
Schematic summary of the growth and salt tolerance mechanisms triggered by the plant growth-promoting endophytic *Streptomyces* sp. GKU 895 inoculated in salt-susceptible rice cultivar IR29 (*Oryza sativa* L. cv. IR29) (**a**). Heat map for expression of DEGs involved in growth and development (**b**), plant hormones (**c**), ion homeostasis (**d**), antioxidants and plant–microbe interactions (**e**). NSC, non-salt control (0 mM NaCl); NSB, non-salt GKU 895 inoculation; SC, salt-stress control (150 mM NaCl); SB, salt-stress GKU 895 inoculation. Arrow, positive regulation; dashed arrow, indirect positive regulation; bar headed line, inhibition

The expression of salt-responsive genes altered by endophytic *Streptomyces* was suggested based on TFs and functional annotation, which included growth and development, plant hormones, ion homeostasis, antioxidants, and plant–microbe interactions, as shown in Fig. 4a. In this study, DEGs involved in photorespiration and calvin cycle pathways were downregulated under salt-stress conditions including those genes involved in photosynthesis such as chlorophyll a-b binding protein (*Chla/b2*; LOC_Os09g17740) and ribulose 1,5- bisphosphate carboxylases/oxygenase (RuBisCO; LOC_Os12g19470 and LOC_Os03g07300), but slightly reduced in IR29 inoculated with *Streptomyces* sp. GKU 895 (Fig. 4b, Additional file 2: Table S3). Moreover, genes responsible in phenylpropanoid and suberin pathways that functioned in cell wall biosynthesis such as phenylalanine ammonia-lyase (LOC_Os02g41630) was upregulated in GKU 895 inoculated salt-stressed rice (SB/NSB). Remarkably, *MAPK5* (LOC_Os03g17700) involved in multiple stress responses was exclusively upregulated in GKU 895 inoculated salt-stressed IR29 (SB/NSB) suggesting that *Streptomyces* sp. GKU 895 enhanced salt tolerance by activation of salt-responsive MAPK cascade genes.

Genes playing role in plant hormones composed of abscisic acid (ABA), auxin (indole-3-acetic acid; IAA), and ethylene, were represented in SB/NSB at higher numbers than other treatments (Fig. 4c, Additional file 2: Table S3). Transcriptional activators of the ABA-inducible genes such as *ABF4*, *OsBZIP12* (LOC_Os12g13170, LOC_Os02g52780, LOC_Os01g64000, LOC_Os05g41070, and LOC_Os03g21800), and NAC transcription factor 105 (LOC_Os08g01330 and LOC_Os08g44820) were positively triggered resulting in upregulation of the key ABA biosynthesis genes, 9-cis-epoxycarotenoid dioxygenase 4 (*OsNCED4,* LOC_Os07g05940) and abscisic acid 8'-hydroxylase 1 (*OsABA8ox1*, LOC_Os02g47470), in GKU 895 inoculated salt-stressed IR29. DEGs of TFs involved in auxin and ethylene including auxin response factor 21 (*OsARF7b*, LOC_Os08g40900) and several genes in ethylene pathway including ACC synthase 1 (*ACS1*, LOC_Os03g01710), ACC oxidase 1 (*ACO1*, LOC_Os02g01710), ethylene responsive element binding protein (EREBP)-like protein (LOC_Os09g28440), ethylene intensive like protein 2 (*EIL2*, LOC_Os07g48630) and WRKY78 (LOC_Os07g39480) responding to *ACO1* were upregulated in salt-stressed IR29 (Fig. 4c, Additional file 2: Table S3). However, two key genes involved in ethylene, *ACO1* and EREBP-like protein, were downregulated in rice inoculated with strain GKU 895. These results suggest that *Streptomyces* sp. GKU 895 positively regulated transcriptional activators that control ABA and auxin biosynthesis, but negatively regulate genes of ethylene biosynthesis.

A number of ion transport and homeostasis related genes such as aquaporin, calmodulin, and potassium channel were detected during salt-stress treatment in rice IR29 with or without inoculation of *Streptomyces* sp. GKU 895. Remarkably, aquaporin (LOC_Os01g38980), *CAM* (LOC_Os01g72009), V-type proton ATPase (V-ATPase) (LOC_Os12g07140), and chloride channel proteins (LOC_Os12g25200 and LOC_Os02g07160), were highly upregulated in GKU 895 inoculated plants under salinity (Fig. 4d, Additional file 2: Table S3). Moreover, many DEGs encoding for antioxidants that function as reactive oxygen species (ROS) scavenger were also altered in salt treated IR29 by *Streptomyces* sp. GKU 895. These included genes related to antioxidative enzymes such as catalase (*CAT*; LOC_Os01g74410), glutathione-S-transferases (*GST*; LOC_Os01g72140 and LOC_Os03g50960), and superoxide dismutase (*SOD*; LOC_Os02g12480) that were upregulated in salt-stressed plants, but downregulated in the present of the endophyte (Fig. 4e, Additional file 2: Table S3). Further investigation on DEGs involved in plant–microbe interactions revealed that pathogenesis-related (PR) protein Bet v (LOC_Os12g36830) and symbiosis-related protein-like protein (LOC_Os07g46380) were expressed only in GKU 895 inoculated rice, while *RPR10a* was downregulated (Fig. 4e, Additional file 2: Table S3).

### Validation of the RNA-Seq and Gene Expression Analysis by RT-qPCR

Among these, one gene involved in the growth and development, *Chla/b2*, three genes in plant hormone pathways (*ACO1*, *OsARF7b* and *OsNCED4*), one gene related to antioxidants and compatible solutes (*CATb*), and one gene associated with ion homeostasis (*Cam1-1*) were selected to validate gene expression by RT-qPCR. The results indicated that the expression pattern of all selected genes by RT-qPCR were similarly as observed from RNA-Seq data (R^2^ = 0.8339) (Additional file 7: Fig. S1), suggesting that the validation analysis supports the reliability of the relative values provided by the transcriptome data.

## Discussion

Plants respond to salt stress with changes in several physiological and metabolic processes (Gupta and Huang 2014). Besides promoting the growth of sugarcane under individual and co-inoculation with endophytic diazotrophs (Kruasuwan and Thamchaipenet 2016), our data show that the endophytic *Streptomyces* sp. GKU 895 promoted the growth of the salt-sensitive rice IR 29 with and without salinity. This suggests that *Streptomyces* sp. GKU 895 may colonize a wider range of plant hosts and confer benefits under different conditions including abiotic stresses. Several genes encoding for PGP-traits, especially bacterial stress responsive genes including ACC deaminase, proline dehydrogenase, superoxide dismutase, and trehalose synthase were discovered in genome of *Streptomyces* sp. GKU 895 (Kruasuwan et al. 2017). Additionally, strain GKU 895 was capable on growth at high salt concentration up to 5% NaCl (Additional file 7: Fig. S2). Recently, the increased growth of rice by a PGPE through nutrient uptake was evidenced by the enrichment of ^15^N in rice inoculated with *Herbaspirillum seropedicae* (Ramos et al. 2020). In our study, endophytic *Streptomyces* sp. GKU 895 was further proved to promote growth by increasing plant biomass (roots and shoots) as well as enhance salt tolerance in a different plant host like *Oryza sativa* L. cultivar IR29. Salinity and PGPE inoculation influenced nutrient uptake in IR29 by enriching a cellular nitrogen compound metabolic process GO term in a common 75 upregulated DEGs across SB/SNB and SC/NSC. Our results are agree with other studies suggested that salt stress has an impact on nitrogen metabolism of old and young leaves in rice (Wang et al. 2012) and enhances gene expression related to nutrient acquisition in wheat colonized with *Azospirillum brasilense* (Camilios-Neto et al. 2014). Salinity altered translation (GO: 0006412) and cellular biosynthetic process (GO: 0044249) GO terms in downregulated DEGs of SC/NSC. In the presence of *Streptomyces* sp. GKU 895, plant genes related to aromatic amino acid family metabolic process (GO: 0009072) and ion transport (GO: 000681) were upregulated. Our results show that *Streptomyces* sp. GKU 895 was able to: (i) improve growth of salt-susceptible rice cultivar IR29 by altering the expression of genes involved in nutrient metabolism; and (ii) alleviate salt tolerance by enhancing ion transport between inner and outer plant cells.

Several TF families related to stress response such as ARF, ERF, and WRKYs were differentially expressed in rice IR29 under salinity and *Streptomyces* sp. GKU 895 inoculation. The results were similar to the transcriptome analysis of salt-sensitive rice IR64 inoculated with an endophytic fungal *Fusarium* sp. isolated from salt-tolerant Pokkali rice that stress-responsive TFs such as bHLH, bZIP, MYB, and WRKY proteins were enriched in salt-exposed and endophyte-colonized seedlings (Sampangi-Ramaiah et al. 2020). Recently, qRT-PCR of *SmARF* gene in eggplant has demonstrated that ARF quickly responded to salt stress (Shen et al. 2022). In agreement, we observed that ARF (LOC_Os01g70270), a key TF response to auxin and various abiotic stresses, was enriched in downregulated DEGs of salt-stressed rice which possibly resulted in growth retardation. Ethylene-responsive factors (ERFs) are important for regulating plant growth, development, and abiotic stress response. Overexpression of *SmERF1* acted as a positive regulator of eggplant response to salt stress (Shen et al. 2022). Hence, enrichment of ERF (*OsERF12*) in this study suggested that *Streptomyces* sp. GKU 895 altered the positive role of such TF in rice towards salt-stress tolerance. Moreover, significant expression of TFs ERF, C2H2, bHLH, GRAs, and WRKYs responding to the stress of low temperature and ultraviolet radiation were also found in transcriptome profiling of the grass, *Stipa purpurea,* inhabited with *Bacillus subtilis* which implied function of resistance genes in *S. purpurea* (Jin et al. 2022).

Under salinity, genes involved in phenylpropanoid and suberin biosynthesis were only induced in salt-stressed rice inoculated with *Streptomyces* sp. GKU 895. Phenylpropanoid pathway, mainly responsible for lignin biosynthesis and plant secondary metabolites, was reported to prevent salinity stress in *Brassica juncea*, Chinese cabbages, and *Sophora alopecuroides* (Wani et al. 2013; Cao et al. 2020; Zhu et al. 2021). Suberin, a cell-wall-associated hetero-polymer, was reported to control salt translocation to the shoot and reduced water loss in *Arabidopsis* (de Silva et al. 2021) and was developed by ABA induction (Wei et al. 2020). Furthermore, genes related to chlorophyll biosynthesis (*Chla/b2*) and RuBisCO were downregulated in salt-stressed rice IR29 which consequently caused degradation in photosynthetic pigments and reduction of PSII activity leading to growth inhibition and yield loss in rice (do Amaral et al. 2016; Razzaque et al. 2017). In our study, inoculation with *Streptomyces* sp. GKU 895 was able to prevent salinity to slightly downregulate those genes in IR29, which allowed to maintain the expression of those genes, and their respective processes. However, these genes were highly expressed when *Arabidopsis* was inoculated with *Bacillus amyloliquefaciens* FZB42 under salinity (100 mM NaCl) (Liu et al. 2017). It is suggested that different type of plants and PGPEs as well as level of salinity may affect the expression of such photosynthesis genes.

In this study, a transcriptional network associated with stress responses SNAC1 was upregulated in salt-stressed rice (SC/NSC) and rice inoculated with *Streptomyces* sp. GKU 895 (NSB/NSC). SNAC1 was positively regulated the expression of ABA signalling genes in rice responding to water loss and stress responses (Li et al. 2019). Genes involved in plant hormones including ABA, auxin, and ethylene as well as their TFs in rice IR29 were differentially regulated by strain GKU 895 in response to salinity. Increment of gene expression of ABA-transcriptional activators, *ABF4*, *OsBZIP12*, and *NAC105* in salt-stressed rice IR29 with or without strain GKU 895 consequently altered ABA biosynthesis by upregulation of *OsNCED4*, a key enzyme for ABA production as well as *OsABA8ox1*. *OsABA8ox1* and *OsNCED4* are one of the determinants of endogenous ABA levels affecting numerous aspects of plant growth and abiotic stress responses which acting as positive regulators of salt tolerance (Zou et al. 2008; Uno et al. 2000). Such overexpression of *NCED* was previously noted to improve drought and salinity in tobacco and creeping bentgrass (Zhang et al. 2008; Aswath et al. 2005). In this study, auxin response factor 21 (*OsARF7b*) was also upregulated in salt-stressed IR29 but higher expressed in the presence of *Streptomyces* sp. GKU 895. Likewise, transcriptomic data of salt-stressed rice treated with fungal endophyte *Piriformospora indica* indicated that genes associated with auxin-activated signalling pathway were mainly enriched, particularly, auxin-responsive Aux/IAA gene family which implied the coordination between hormone and root/shoot length contributing to the better growth in rice (Nivedita et al. 2020). IAA is an auxin-related plant hormones that plays major role in plant growth and stress response (Emenecker and Strader 2020). Several auxin-response factors encoding genes were relatively repressed when rice and tomato were treated with salt (Bouzroud et al. 2018; Jain and Khurana 2009).

Here, *MAPK5* was upregulated in salt-stressed inoculated rice (SB/NSB) together with ERF. Ethylene is one of the plant hormones that mediates response to stresses including salinity (Riyazuddin et al. 2020) and also mediates crosstalk between mitogen activated protein kinase (MAPK) signaling pathways (Ludwig et al. 2005). Similarly, the bacterial endophytes *Bacillus* induced the expression of MAPK signaling and *WRKY53* gene in order to activate resistance against necrotrophic pathogen in *Arabidopsis* (Nie et al. 2017). Moreover, ACC deaminase-producing *Enterobacter*, *Bacillus*, *Brevibacterium*, and *Streptomyces* were reported to reduce ethylene in response to salinity in Arabidopsis, tomato, red pepper, and rice by activity of ACC deaminase that converted ACC to ammonia and α-ketobutyrate and subsequently reduced level of stress ethylene (Siddikee et al. 2011; Kangmin et al. 2014; Yoolong et al. 2019; Kruasuwan and Thamchaipenet 2018; Jaemsaeng et al. 2018). In this study, key genes in ethylene biosynthesis, *ACO1*, *EIL2*, *EREBP1*, and a transcription factor WRKY78 that controls *ACO1*, were upregulated in salt-stressed IR29 but downregulated in the presence of *Streptomyces* sp. GKU 895. EREBP is a member of the ethylene-response factor (ERF) family, which functions downstream of *EIN3* and plays an important role in abiotic and biotic stress responses (Chen et al. 2003; Riyazuddin et al. 2020). High salt concentrations induced the accumulation of EIN3/EIL proteins in *Arabidopsis* and increased *EREBP1* expression in tobacco (Peng et al. 2014). Similar to this findings, the expression of *ERF* was reduced when canola plants was inoculated with ACC-deaminase producing *Pseudomonas putida* UW4 (ACD^+^) compared with ACD^–^ strain (Stearns et al. 2012). Hence, ACC deaminase-producing *Streptomyces* sp. GKU 895 reduced the expression of those ethylene-related genes in rice grown under salinity through the consumption of ACC and consequently lower the plant ethylene.

Salinity also adversely affects plant growth by causing high internal Na^+^ accumulation and disrupting plant potassium nutrition which is a common plant response to salt-stress conditions (Zhu 2001). Controlling ionic homeostasis is considered an important mechanism for salinity tolerance. Based on transcriptome analysis of this study, several genes encoding aquaporin, calmodulin, and potassium transporter were upregulated in salt-stressed rice IR29 with or without inoculation of *Streptomyces* sp. GKU 895. Aquaporins are channel proteins is considered an important role in plant water relations that influences plant growth and involved in plant response to stresses (Kapilan et al. 2018; Shekoofa and Sinclair 2018). Consistently to the previous report, aquaporin was upregulated in wheat inoculated with *Arthrobacter nitroguajacolicus* under 200 mM NaCl stress (Shekoofa and Sinclair (2018). Calcium signalling network is one of the signal cascades involved in transient changes in cytosolic Ca^2+^ concentration, which was reported to be a key messenger in the salt-stress response (Mahajan and Tuteja 2005). The binding of Ca^2+^ to the calmodulin complex, encoded by *CaM 1-1*, regulates a variety of cellular processes implicated in salt and other stresses (Kim et al. 2009). Therefore, *Cam1-1* gene is a significant player in the Ca^2+^ signal transduction network. In this experiment, *CAM* (LOC_Os01g72009) of IR29 was upregulated under salt-stress condition and highest in plants inoculated with *Streptomyces* sp. GKU 895 suggesting that strain GKU 895 plays a positive role to induce calmodulin to help rice plants tolerate to salinity. Similar result was reported in plant growth-producing *Streptomyces* increasing salt tolerance in rice by upregulation of *Cam1-1* (Jaemsaeng et al. 2018). Furthermore, overexpression of *OsCam1-1* induced ABA biosynthesis by upregulation of *NCED3* in Thai rice cultivar KDML105 under salinity stress (Saeng-ngam et al. 2012) which was correlated with the findings in this study. In addition, a major functional for H^+^ pump, V-ATPase, and chloride channel protein that plays a role in mediation of Cl^–^ transportation, were highly expressed in salt-stressed rice associated with *Streptomyces* sp. GKU 895. The results supported by other reports that expression of vacuolar H^+^‐ATPase subunit c1 gene (*SaVHAc1*) of halophyte grass could enhance salt tolerance of rice plants (Baisakh et al. 2012) and overexpression of *GmCLC1* enhanced salt tolerance in transgenic *Arabidopsis thaliana* by reducing the Cl^–^ accumulation in shoots (Wei et al. 2016). The findings suggested that *Streptomyces* sp. GKU 895 alleviates salt tolerance of IR29 by uptake or release of either H^+^ or Cl^–^ in order to balance the excess of Na^+^ in the plant cells and, hence, released the negative impact of salt stress.

Salinity causes oxidative stress which then promotes ROS accumulation like super oxide radicals (O^2−^) and hydrogen peroxide (H_2_O_2_) that are potentially harmful to cell integrity and lead to growth inhibition (Munns and Tester 2008). To deal with the adverse effects of oxidative stress, plant cells are mitigated by a large number of antioxidant enzymes such as catalase (CAT), glutathione-S-transferase (GST), lipoxygenase (LOX), L-ascobate oxidase (APX), and superoxide dismutase (SOD) (Choudhury et al. 2017). Recent report showed that *Bradyrhizobium* co-inoculated with either *Bacillus* or *Paenibacillus* significantly increased SOD and total ascorbate in both non-salt and salt-stressed cowpea (Santos et al. 2018). Furthermore, genes encoding ROS scavenging antioxidative enzymes such as *APX*, *CAT*, *SOD*, and *GST* were upregulated in barley and rice under salinity stress (Baltruschat et al. 2008; Vighi et al. 2017). Those reports were in agreement with this study that *CAT*, *GST* and *SOD* were highly expressed in salt-stressed rice IR29 but were downregulated in the presence of *Streptomyces* sp. GKU 895. Similar to RNA-seq profiling of rice seedling inoculated with endophytic fungi, *Sordariomycetes* sp. EF0801, under Na_2_CO_3_ treatment that genes related to ROS-scavenging systems, *APX1*, *CATA*, and *CATB* as well as TFs of bZIP family involved in plant hormone signalling were upregulated (Ren et al. 2022). CAT, GST, and SOD are considered as an alarm in salt stress response in plants. SOD catalyses the dismutation of O_2_^–^ to H_2_O_2_ and molecular oxygen, CAT then protects the cell from H_2_O_2_ by catalysing its decomposition into O_2_ and H_2_O (Dionisio-Sese and Tobita 1998). Downregulation of those genes, therefore, suggested that inoculation of *Streptomyces* sp. GKU 895 may reduce stress level in rice plants.

Beneficial plant–microbe interactions have been rarely studied for locally induced changes in plant gene expression (Van Wees et al. 2008). In our experiment, genes involved in plant–microbe interaction regarding to pathogenesis-related (PR) protein Bet v I and symbiosis-related protein-like protein (LOC_Os07g46380) were solely expressed in GKU 895 inoculated rice plants. Pathogenesis-related Bet v I belongs to family 10 of plant PR protein that plays important role in the defence response to biotic stress (Liu and Ekramoddoullah 2006). *RPR10a* transcript was documented to highly express in rice infected with a fungal pathogen, *Magnaporthe grisea* (McGee et al. 2001). In contrast, this gene was downregulated in non-salt rice IR29 inoculated with *Streptomyces* sp. GKU 895. The results suggested that *Streptomyces* sp. GKU 895 colonized the rice plant with no harm and may trigger plant immune system.

## Conclusion

Our data show that the plant growth-promoting endophytic *Streptomyces* sp. GKU 895 carrying ACC deaminase can enhance growth and improve salt tolerance of a salt-susceptible rice cultivar. Therefore, this endophyte can be used to confer salt tolerance in another agriculturally important plant like rice. Transcriptome profiling indicated that *Streptomyces* sp. GKU 895 induced the upregulated expression of several key genes and their corresponding transcription factors that play important roles in growth and development, photosynthesis, plant hormones, ROS scavenging, ion transport and homeostasis, to confer salt tolerance in plant. The beneficial effects will lead to essential applications of the environmentally friendly endophyte in the actual field which may facilitate growth with reasonable yields of salt-sensitive crops under marginal saline lands without any traditional or genetic engineering plant improvement.

## Materials and Methods

### Plant Growth and Bacterial Inoculation

Salt-susceptible rice cultivar IR29 (*Oryza sativa* L. cv. IR29) seeds were obtained from Nakhon Ratchasima Rice Research Centre (Nakhon Ratchasima, Thailand). Seeds were surface sterilised with 70% ethanol for 5 min following by 2% sodium hypochlorite for 45 min and washed thoroughly in sterile distilled water. The seeds were then placed on sterilised water saturated tissue papers in plastic trays for 7 days in the dark. Seven-day-old rice seedlings were root dipped with spore suspension of *Streptomyces* sp. GKU 895 (10^8^ spores ml^−1^) for 4 h. Seedlings were cultured hydroponically in a half-strength Yoshida culture solution (YS) (Yoshida et al. 1976) for 7 days, then, transferred to full-strength YS for 7 days under greenhouse condition. After 14 days, rice seedlings were subjected to salt-stress treatment (150 mM NaCl). After 7 days post irrigation (dpi), seedlings were collected and immediately snap-frozen with liquid nitrogen and keep at −80 °C until use. The experiment was set up in completely randomised design (CRD) using ten replicate plants per treatment and each treatment was independently replicated twice.

### Plant Growth Measurement and ROS Straining

Growth parameters, root/shoot lengths and dry weights, of rice plants at 7 dpi were measured. For superoxide and hydrogen peroxide staining, were detected by immersion of rice leaves in 25 mL of nitrobluetetrazolium solution (NBT; 0.5 µg mL^−1^ NBT in 10 mM phosphate buffer, pH 7.6) for 3 h in the dark and in 25 mL of 3,3′-diaminobenxidine solution (DAB; 1 µg mL^−1^ in 50 mM Tris–acetate buffer, pH 5.0) for 8 h, respectively. After staining, both treatments were boiled in 95% (v/v) ethanol for 30 min and subsequently immersed in 40% glycerol for 16 h before colour detection.

### RNA Isolation, Library Preparation, and Ion PGM Sequencing

Two independent experiments were conducted for RNA-seq analysis. Ten plants of each replicate of both inoculated and un-inoculated treatments were used for total RNA extraction. Total RNA from ten individual plants was prepared by CTAB method according to Yu and Goh (2000). The quantity and quality of RNA samples were determined by Nanodrop^®^ spectrophotometer and Agilent^®^ RNA 6000 Pico Kit with Agilent^®^ 2100 Bioanalyzer^®^. Total ribosomal RNAs (rRNA) were thoroughly depleted by Ribo-Zero Magnetic Kit for plant seeds/roots and Gram-positive bacteria (Illumina Inc., USA). Library was constructed as described in the Ion Total RNA-Seq Kit v2 library preparation guide (Thermo Fisher Scientific, USA) by preparing template-positive using Ion PGM™ Hi-Q™ OT2 kit (Thermo Fisher Scientific, USA) and enriching using Ion OneTouch™ ES Kit (Thermo Fisher Scientific, USA) according to the manufacture protocol. The cDNA libraries were sequenced by Ion PGM™ sequencer using Ion 316™ chip.

### Transcriptome Data Analysis

All RNA-seq reads were analysed using The Discovery Environment (DE, https://de.cyverse.org/de/) in the CyVerse server. The reads were qualified by FastQC and mapped against the rice genome (*Oryza sativa* IRGSP-1.0; BioProject number PRJDB1747) using HISAT2 version 1.0 (Kim et al. 2015). The mapped reads were assembled and merged using Cufflinks package (Trapnell et al. 2012) and differentially expressed genes (DEGs) of treatments were calculated by Cuffdiff (Trapnell et al. 2012). Venn diagrams of pair-wise overlap between the treatments and control of up and downregulated DEGs were analysed by Venny version 2.0.2 (Oliveros 2007–2015). To gain further insight into the biological processes underpinning salt tolerance in the salt-susceptible rice IR29 inoculated with and without *Streptomyces* sp. GKU 895, the DEGs from all treatments were subjected to gene ontology (GO) analysis to assign these to particular biological processes using agriGO version 2.0 (Du et al. 2010). A GO category was accepted under a hypergeometric test *p* < 0.05 with false discovery rate (FDR) < 0.05 and Log_2_ fold change (FC) of ≥ 1.5 and ≤ 1.5 as up and downregulation, respectively. Pathway assignments were performed using Plant Reactome (https://plantreactome.gramene.org; Naithani et al. 2019). Plant transcription factors were determined using PlantTFDB 4.0 (Jin et al. 2017). Hierarchical cluster analysis and heatmaps of DEGs and GO terms were generated using gplot the R package (version 3.4.0).

### Real-time PCR Analysis of Genes Involved in Salt-Stress Response

Total RNA was treated with DNase I (Thermo Fisher Scientific, USA). cDNA was synthesized using the Thermo Scientific RevertAid First strand cDNA synthesis Kit (Thermo Fisher Scientific, USA). KAPA SYBR^®^ FAST qPCR Master Mix (2 ×) (Kapa Biosystem Inc., Korea) was used for quantification in Master Cycler Realplex 4 (Eppendorf, USA). Six genes involved in growth and salt tolerance were randomly chosen based on biological process categories to validate the levels of expression using quantitative real-time PCR (RT-qPCR) (Additional file 1: Table S8). The mean value was calculated from three replicates and normalized with actin (*ACT*) as an internal control.

### Statistical Analysis

The biological and technical triplicate data of plant growth parameters were statistically evaluated with two-way ANOVA for the linear model on raw data followed by Turkey’s test (***p < 0.001). Mean ± SD values with different superscript letters indicate statistical differences (*p* < 0.05) analysed by IBM^®^ SPSS^®^ statistic version 28 (IBM Inc., USA).

## Supplementary Information

**Additional file 1: Table S1.** Shoot and root dry weights of salt-susceptible rice cultivar IR29 (*Oryza sativa* L. cv. IR29) conferred by plant growth-promoting endophytic *Streptomyces* sp. GKU 895. **Table S2.** Qualification of raw read sequences and mapped read percentage aligned against rice genome (*Oryza sativa* IRGSP-1.0). **Table S8.** RT-qPCR primers used in this study.

**Additional file 2: Table S3.** List and predicted function of up and downregulated DEGs.

**Additional file 3: Table S4.** List and GO terms analysis of common up and downregulated DEGs.

**Additional file 4: Table S5.** List of gene and GO term analysis of exclusive up and downregulated DEGs.

**Additional file 5: Table S6.** Metabolic pathway analysis of up and downregulated DEGs.

**Additional file 6: Table S7.** Transcription factor (TF) related gene analysis of up- and downregulated DEGs.

**Additional file 7: Fig. S1.** Linear regression analysis indicated a correlation between the Log_2_ (Fold change; FC) of RNA-seq and RT-qPCR. NSC, non-salt control (0 mM NaCl); NSB, non-salt GKU 895 inoculation; SC, salt-stress control (150 mM NaCl); SB, salt-stress GKU 895 inoculation. **Fig. S2.** Salt tolerance of *Streptomyces* sp. GKU 895 and salt-susceptible rice cultivar IR29 (*Oryza sativa* L. cv. IR29); (a) GKU 895 on ISP2 agar supplemented with 1%, 3%, 5%, and 7% NaCl for 7 days; (b) IR29 under hydroponic condition containing 50, 100, 150, and 200 mM NaCl for 7 days.

## Data Availability

All raw read sequences retrieved from Ion Torrent were deposited to GenBank database as a sequence read archive (SRA) accession number SRP126983 in the BioProject number PRJNA422335.
