# Supplementary material for: Transcriptome Landscapes of Salt-Susceptible Rice Cultivar IR29 Associated with a Plant Growth Promoting Endophytic Streptomyces
Source: Rice (N Y). 2023 Feb 4;16:6. doi: 10.1186/s12284-023-00622-7 (PMC9899303; doi:10.1186/s12284-023-00622-7)
Supplement: Supplementary file 1 — Additional file 1: Table S1. Shoot and root dry weights of salt-susceptible rice cultivar IR29 (Oryza sativa L. cv. IR29) conferred by plant growth-promoting endophytic Streptomyces sp. GKU 895. Table S2. Qualification of raw read sequences and mapped read percentage aligned against rice genome (Oryza sativa IRGSP-1.0). Table S8. RT-qPCR primers used in this study. [file 12284_2023_622_MOESM1_ESM.docx]

**Additional file: Tables**

**Transcriptome landscapes of salt-susceptible rice cultivar IR29 associated with plant growth promoting endophytic *Streptomyces***

Worarat Kruasuwan^1,2,5^, Karan Lohmaneeratana^1^, John T. Munnoch^2^,

Wanwipa Vongsangnak^3,4^, Chatchawan Jantrasuriyarat^1^, Paul A. Hoskisson^2^,

and Arinthip Thamchaipenet^1,4,^*

^1^Department of Genetics, Faculty of Sciences, Kasetsart University, Bangkok, Thailand

^2^Strathclyde Institute of Pharmacy and Biomedical Sciences, University of Strathclyde, Glasgow, United Kingdom

^3^Department of Zoology, Faculty of Sciences, Kasetsart University, Bangkok, Thailand

^4^Omics Center for Agriculture, Bioresources, Food and Health, Kasetsart University (OmiKU), Bangkok, Thailand

^5^Present address: Siriraj Long-read Lab (Si-LoL), Division of Medical Bioinformatics, Research Department, Faculty of Medicine Siriraj Hospital, Mahidol University, Bangkok, Thailand

***Corresponding author:**

Arinthip Thamchaipenet

E-mail address: arinthip.t@ku.ac.th

**Additional file 1:** **Table S1**. Shoot and root dry weights of salt-susceptible rice cultivar IR29 (*Oryza sativa* L. cv. IR29) conferred by plant growth-promoting endophytic *Streptomyces* sp. GKU 895

| **Treatment** | **Dry weight (mg)** | |
| --- | --- | --- |
|  | **Shoot** | **Root** |
| NSC | 103.73 ± 24.5^b^ | 22.55 ± 7.79^b^ |
| SC | 61.80 ± 36.89^d^ | 15.85 ± 4.98^c^ |
| NSB | 154.30 ± 15.44^a^ | 33.90 ± 17.75^a^ |
| SB | 99.50 ± 23.18^c^ | 28.93 ± 17.24^a^ |

**Note:** NSC, non-salt control (0 mM NaCl); NSB, non-salt GKU 895 inoculation; SC, salt-stress control (150 mM NaCl); SB, salt-stress GKU 895 inoculation.

**Additional file 1: Table S2.** Qualification of raw read sequences and mapped read percentage aligned against rice genome (*Oryza sativa* IRGSP-1.0)

| **Sample** | **Replicates** | **Raw reads (bp)** | **Mapping reads (%)** |
| --- | --- | --- | --- |
| NSC | 1 | 2,215,837 | 1,841,139 (83.09) |
|  | 2 | 2,036,986 | 1,636,718 (80.35) |
| SC | 1 | 2,027,307 | 1,630,968 (80.45) |
|  | 2 | 1,596,990 | 1,262,580 (79.06) |
| NSB | 1 | 2,125,536 | 1,672,584 (78.69) |
|  | 2 | 2,506,691 | 1,575,293 (78.72) |
| SB | 1 | 2,109,422 | 1,813,259 (85.96) |
|  | 2 | 1,354,164 | 1,128,154 (83.31) |

**Note:** NSC, non-salt control (0 mM NaCl); NSB, non-salt GKU 895 inoculation; SC, salt-stress control (150 mM NaCl); SB, salt-stress GKU 895 inoculation.

**Additional file 1: Table S8**. RT-qPCR primers used in this study

| **Gene** | **Sequences (5'-3')** | **Reference** |
| --- | --- | --- |
| **Growth and developments** | |  |
| Chlorophyll a-b binding protein 2 (*Chla/b2*; LOC_Os09g17740) | F: GCTACCTCACCGGCGAGTT | This study |
|  | R: CAGGCTCGGGTTGCCGA |  |
| **Ethylene biosynthesis** | |  |
| ACC oxidase  (*ACO1*) | F: GATAGCGTGTGTACCACAGCGACC | (Iwai et al, 2006) |
|  | R: CACGGTACAGCACGCCGCAC |  |
| **Hormones** | |  |
| Auxin-responsive protein  (*OsARF7b*; LOC_Os08g40900) | F: TACATCGTACACATTTCACAAAGTC | This study |
|  | R: GGAGGATAACCGGCGACTAG |  |
| 9-cis-epoxycarotenoid dioxygenase (*OsNCED4*; LOC_Os07g05940) | F: TCCATCTCCTTCTCCCTCCTCCCA | This study |
|  | R: CCTCGCACCCTGCTTGATCTTGCC |  |
| **Antioxidant and compatible solutes** | |  |
| Catalase (*CATb*) | F: ATGGATCCCTACAAGCATCG | (Nautiyal et al, 2013) |
|  | R: GGCTCCCCTTGCATGAACGAC |  |
| **Ion homeostasis** | |  |
| Calmodulin (*Cam1-1*) | F: ACCGTGCATTGCCGTATTAG | (Chinpongpanich et al, 2012) |
|  | R: GCAAGCCTTAACAGATTCAC |  |
| **Housekeeping genes** |  |  |
| Actin (*ACT1*) | F: AGCTATCGTCCACAGGAA | (Saeng-ngam  et al, 2012) |
|  | R: ACCGGAGCTAATCAGAGT |  |

Chinpongpanich A, Limruengroj K, Phean-o-pas S, Limpaseni T, Buaboocha T (2012). Expression analysis of calmodulin and calmodulin-like genes from rice, *Oryza sativa* L. BMC Res Notes 5:625

Iwai T, Miyasaka A, Seo S, Ohashi Y (2006). Contribution of ethylene biosynthesis for resistance to blast fungus infection in young rice plants. Plant Physiol 142:1202–1215

Nautiyal CS, Srivastava S, Chauhan PS, Seem K, Mishra A, Sopory SK (2013). Plant growth-promoting bacteria *Bacillus amyloliquefaciens* NBRISN13 modulates gene expression profile of leaf and rhizosphere community in rice during salt stress. Plant Physiol. Biochem. 66:1–9

Saeng-ngam S, Takpirom W, Buaboocha T, Chadchawan S (2012). The role of the *OsCam1-1* salt stress sensor in ABA accumulation and salt tolerance in rice. J Plant Biol 55:198–208

**References**
