# Supplementary material for: Transcriptome Landscapes of Salt-Susceptible Rice Cultivar IR29 Associated with a Plant Growth Promoting Endophytic Streptomyces
Source: Rice (N Y). 2023 Feb 4;16:6. doi: 10.1186/s12284-023-00622-7 (PMC9899303; doi:10.1186/s12284-023-00622-7)
Supplement: Supplementary file 7 — Additional file 7: Fig. S1. Linear regression analysis indicated a correlation between the Log2 (Fold change; FC) of RNA-seq and RT-qPCR. NSC, non-salt control (0 mM NaCl); NSB, non-salt GKU 895 inoculation; SC, salt-stress control (150 mM NaCl); SB, salt-stress GKU 895 inoculation. Fig. S2. Salt tolerance of Streptomyces sp. GKU 895 and salt-susceptible rice cultivar IR29 (Oryza sativa L. cv. IR29); (a) GKU 895 on ISP2 agar supplemented with 1%, 3%, 5%, and 7% NaCl for 7 days; (b) IR29 under hydroponic condition containing 50, 100, 150, and 200 mM NaCl for 7 days. [file 12284_2023_622_MOESM7_ESM.docx]

**Additional file: Figures**

**Transcriptome landscapes of salt-susceptible rice cultivar IR29 associated with plant growth promoting endophytic *Streptomyces***

Worarat Kruasuwan^1,2,5^, Karan Lohmaneeratana^1^, John T. Munnoch^2^,

Wanwipa Vongsangnak^3,4^, Chatchawan Jantrasuriyarat^1^, Paul A. Hoskisson^2^,

and Arinthip Thamchaipenet^1,4,^*

^1^Department of Genetics, Faculty of Sciences, Kasetsart University, Bangkok, Thailand

^2^Strathclyde Institute of Pharmacy and Biomedical Sciences, University of Strathclyde, Glasgow, United Kingdom

^3^Department of Zoology, Faculty of Sciences, Kasetsart University, Bangkok, Thailand

^4^Omics Center for Agriculture, Bioresources, Food and Health, Kasetsart University (OmiKU), Bangkok, Thailand

^5^Present address: Siriraj Long-read Lab (Si-LoL), Division of Medical Bioinformatics, Research Department, Faculty of Medicine Siriraj Hospital, Mahidol University, Bangkok, Thailand

***Corresponding author:**

Arinthip Thamchaipenet

E-mail address: arinthip.t@ku.ac.th

**
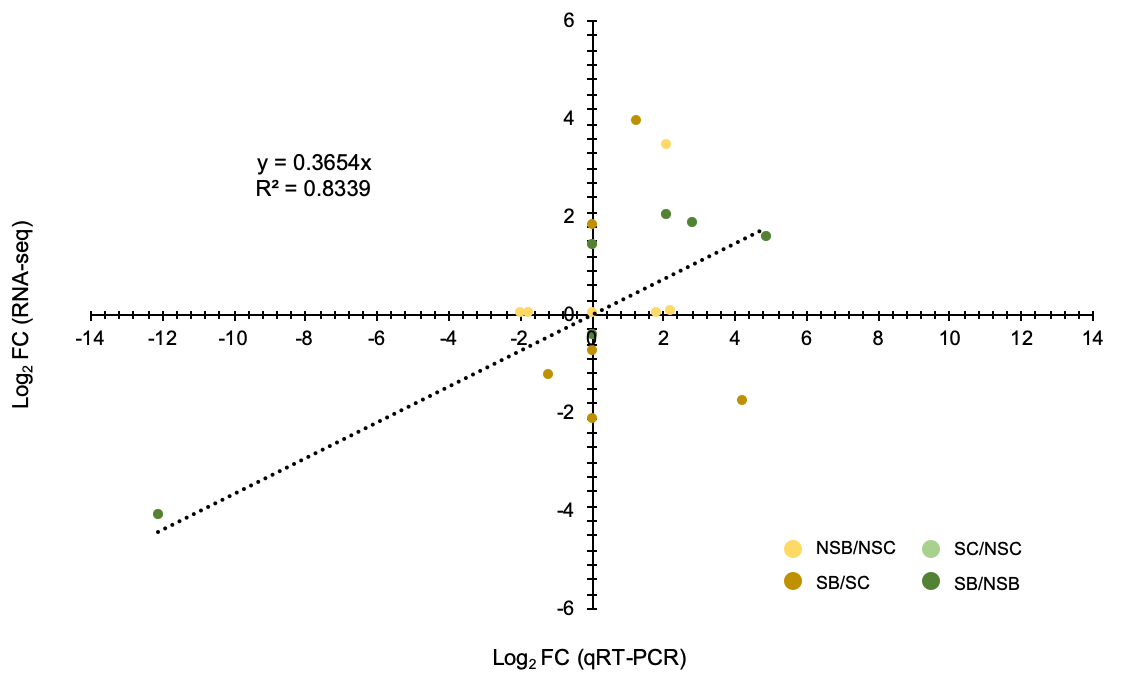
**

**Additional file 7: Fig. S1.** Linear regression analysis indicated a correlation between the Log_2_ (Fold change; FC) of RNA-seq and qRT-PCR. NSC, non-salt control (0 mM NaCl); NSB, non-salt GKU 895 inoculation; SC, salt-stress control (150 mM NaCl); SB, salt-stress GKU 895 inoculation.

**Additional file 7: Fig. S2.** Salt tolerance of *Streptomyces* sp. GKU 895 and salt-susceptible rice cultivar IR29 (*Oryza sativa* L. cv. IR29); (a) GKU 895 on ISP2 agar supplemented with 1%, 3%, 5%, and 7% NaCl for 7 days; (b) IR29 seedlings under hydroponic condition containing 50, 100, 150, and 200 mM NaCl for 7 days.
